# Wearable Polarization Conversion Metasurface MIMO Antenna for Biomedical Applications in 5 GHz WBAN

**DOI:** 10.3390/bios13010073

**Published:** 2023-01-01

**Authors:** Rigeng Wu, Jian Dong, Meng Wang

**Affiliations:** School of Computer Science and Engineering, Central South University, Changsha 410083, China

**Keywords:** metasurface, biomedical, wearable, MIMO, polarization conversion, WBAN

## Abstract

This paper presents a wearable metasurface multiple-input multiple-output (MIMO) antenna for biomedical applications in a 5 GHz wireless body area network (WBAN) with broadband, circular polarization (CP), and high gain. The physical properties of the MIMO antenna element and the principles of polarization conversion are analyzed in-depth using characteristic mode analysis. For the proposed MIMO antenna, the measured −10 dB impedance bandwidth is 34.87% (4.76–6.77 GHz), and the 3 dB axial ratio bandwidth is 22.94% (4.9–6.17 GHz). By adding an isolation strip, the measured isolation of the two antenna elements is greater than 19.85 dB. The overall size of the MIMO antenna is 1.67λ_0_ × 0.81λ_0_ × 0.07λ_0_ at 5.6 GHz, and the maximum gain is 7.95 dBic. The envelope correlation coefficient (ECC) is less than 0.007, with the maximum diversity gain greater than 9.98 dB, and the channel capacity loss is less than 0.29 b/s/Hz. The specific absorption rate (SAR) of the wearable MIMO antenna is simulated by the human tissue model, which proves that the proposed antenna conforms to international standards and is harmless to humans. The proposed wearable metasurface MIMO antenna has CP, broadband, high gain, low ECC, and low SAR, which can be used in wearable devices for biomedical applications.

## 1. Introduction

Nowadays, with the development of wireless communication, wearable antennas are more and more widely used in wireless body area networks (WBANs) [1]. The 5 GHz band, with its wide bandwidth and high data rate, has great potential for development in WBAN and is increasingly becoming the communication band for wearable antenna applications [2]. Due to the requirements of wearable devices, wearable antennas need to have the properties of lightweight, low profile, low loss, easy processing, and stable gain. In terms of biomedicine, the radiation intensity of the wearable antenna to the human body should also be considered, and the back lobe of the antenna is required to be small. Although linearly polarized antennas have been widely used in wearable devices before, it is difficult to maintain good polarization efficiency in a moving human body and to meet the modern complex communication environment. In order to solve the polarization mismatch of wearable devices when the human body is moving, circularly polarized antennas have gradually become a research hotspot in the field of wearable devices [3,4]. The circularly polarized antennas have the characteristics of reducing the multipath effect, polarization mismatch, and absorption loss and are widely used in communication equipment [5,6].

Recently, many wireless communication devices need to transmit and receive data at a high rate, and some multiple-input multiple-output (MIMO) antennas are proposed to be applied to wearable devices [7]. Microstrip patch antennas are widely used in wearable MIMO antennas because of their lightweight, low profile, easy processing, and easy integration. However, with the increasing demand for antennas, microstrip patch MIMO antennas cannot meet modern communication needs due to their narrow bandwidth, high loss, and low efficiency [8,9]. As a two-dimensional form of metamaterial, metasurface has broad application prospects in the field of MIMO antenna design, which can widen the bandwidth, increase the gain, and reduce the size of the antenna [10]. In [11], a two-port MIMO antenna with metamaterial excited reactive impedance surface and electromagnetic band gap (EBG) was proposed. The antenna achieved high isolation of 40 dB in its operating frequency band, but its gain was only 5.8 dBi. In [12], a two-port MIMO antenna for wireless telecommunication and medical systems was proposed. The proposed antenna achieved a wide -10 dB impedance bandwidth (IMBW) of 1.1–8.6 GHz, but no specific absorption rate (SAR) simulation of radiation to the human body was found when the antenna was applied to the wearable device. In [13], a wearable ultra-wideband eight-port MIMO Vivaldi antenna for 5G and satellite communications with operating bandwidths of 3.7–3.85 GHz and 5–40 GHz was proposed. The antenna also achieved a high gain of 10.7 dBi, but the performance parameters of the MIMO antenna had not been fully analyzed. In [14], a two-port wearable MIMO antenna with high isolation and polarization diversity performance was proposed for medical cancer detection applications. The antenna obtained a wide 3 dB axial ratio bandwidth (ARBW) of 37.84%, but the gain of the antenna was small, only 3.45 dBic, and the SAR value was too high, which would cause potential harm to the human body. In [15], a circular polarization (CP) MIMO antenna with polarization/pattern diversity was proposed. The envelope correlation coefficient (ECC) was less than 0.004, with the maximum diversity gain (DG) greater than 9.98 dB, obtaining a MIMO antenna with better performance. The 3 dB ARBW of 18.3% was obtained while achieving low SAR but had a low gain and narrow IMBW. The MIMO antenna mentioned above achieves good MIMO performance, but some antennas have narrow IMBW and ARBW and large backward radiation, which is unfavorable to the human body and will make the antenna performance worse in the moving human body.

In this paper, a broadband CP wearable metasurface MIMO antenna based on characteristic mode analysis (CMA) is proposed. The metasurface unit consists of two symmetrical crowns, and the overall structure is excited by slot-coupled feeding, realizing wide IMBW and ARBW. The conversion principle of linear polarization (LP) to CP is analyzed by CMA, and the resonance characteristics of the proposed MIMO antenna element are comprehensively analyzed. Furthermore, based on the proposed antenna element, a novel wearable metasurface MIMO antenna is designed, which achieves wide IMBW and ARBW and obtains low ECC. At the same time, the 1 g and 10 g SAR values simulated by the MIMO antenna are far lower than international standards and are harmless to the human body.

The rest of the paper is as follows. In Section 2, the slot layer, metasurface layer, and overall structure of the MIMO antenna element are analyzed by CMA, and the conversion principle from LP to CP is fully explained. In Section 3, the full-wave simulation of the overall wearable metasurface MIMO antenna is carried out, and the performance is studied. Simultaneously, the SAR of the wearable MIMO antenna is simulated, and the measurement of the fabricated MIMO antenna is discussed. Section 4 shows the conclusion of this paper.

## 2. MIMO Antenna Element Design and Analysis Based on CMA

**Figure 1:**
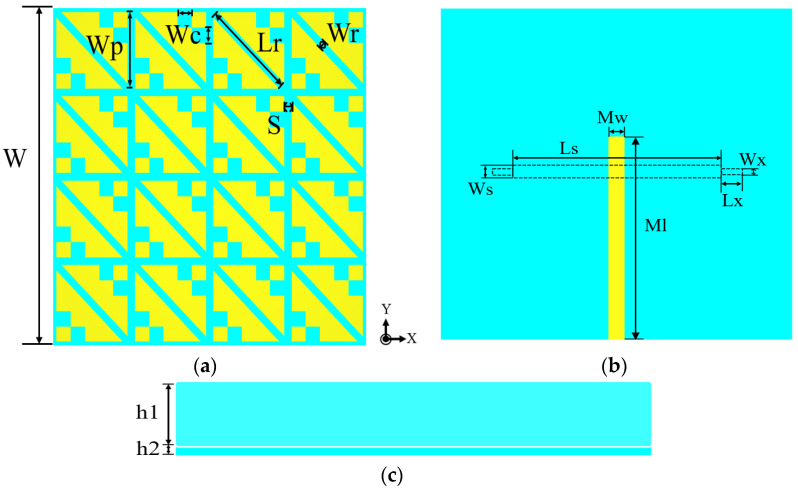
Structure of the MIMO antenna element: (**a**) Front view; (**b**) Back view; (**c**) Side view.

To investigate the working principle of the proposed MIMO antenna polarization conversion, the different layers of the antenna are analyzed using the multi-layer solver in the commercial software CST MWS. The overall structure of the MIMO antenna element is shown in Figure 1. The geometry of the proposed antenna element is a typical sandwich structure consisting of two dielectric substrates and three metal layers. The polytetrafluoroethylene (PTFE) material with stable performance is used as a dielectric substrate with relative permittivity of 2.65 and a loss tangent of 0.0015. The top metal layer is a metasurface layer composed of 4 × 4 symmetrical crown-shaped units, which are used to realize CP radiation, and the stepped slot layer is located between the two dielectric substrates to realize LP radiation; the bottom is the feed structure of a 50 Ω microstrip line. The specific parameters are shown in Table 1.

### 2.1. Analysis of Different Layers Based on CMA

Characteristic mode analysis (CMA) is a popular theory in recent years to analyze the principle of antenna implementation [16,17,18]. It is used to analyze the inherent physical properties of the designed antenna and to achieve the desired function by exciting the ideal mode, which has been widely used and studied by scholars [19,20].

**Figure 2:**
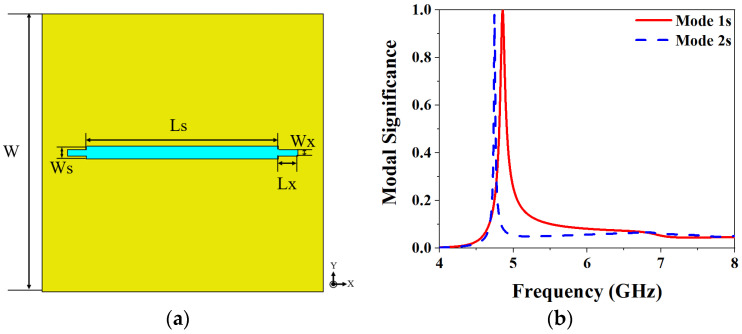
Slot layer structure and CMA results: (**a**) Slot layer structure; (**b**) MS.

**Figure 3:**
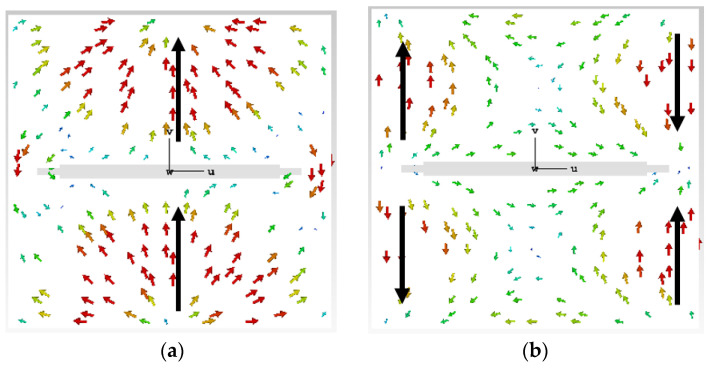
The characteristic current of the slot layer: (**a**) The characteristic current of Mode 1s at 4.8 GHz; (**b**) The characteristic current of Mode 2s at 4.7 GHz.

*CMA-based slot layer analysis.* The first two characteristic modes of the slot layer are taken for CMA in the 4–8 GHz, and the dielectric substrate and ground are set to infinity. The structure of the slot layer is shown in Figure 2a, and the results of CMA are shown in Figure 2b and Figure 3. Modal significance (MS) is used to characterize the potential ability of a characteristic mode, and the calculation formula is as follows:

(1) $${\mathrm{MS}}_{n}=\left|\frac{1}{{1+j\lambda }_{n}}\right|$$

where $\lambda_{n}$ represents the characteristic value (CV) of the n-th mode. If the MS is closer to 1 at a certain frequency, the mode is more important at that frequency, and if the excitation is added in the right place, then this mode will be the dominant radiation mode. From the results of MS in Figure 2b, it can be seen that Mode 1s and Mode 2s are close to 1 at 4.8 GHz and 4.7 GHz, respectively. However, the amplitude of Mode 1s is higher than that of Mode 2s, indicating that Mode 1s has the highest radiation efficiency. Figure 3 shows the characteristic current distributions of the two modes, respectively located at their resonant frequencies. The characteristic current distribution of Mode 1s at 4.8 GHz is mainly along the y-axis, as shown in Figure 3a, but it has less reverse current distribution on both sides of the slot. Due to the small reverse current density, it is difficult to play a dominant role after feeding, so Mode 1s mainly exhibits LP along the y-axis. The characteristic current distribution of Mode 2s is shown in Figure 3b. It can be seen that the current distribution presents an axial symmetry along the x-axis, and the current density distributed on the two sides of the slot antenna is relatively large. If feeding is performed, it is difficult to exhibit the distribution characteristics of LP. So the Mode 1s is chosen as the main radiation mode of the slot layer.

**Figure 4:**
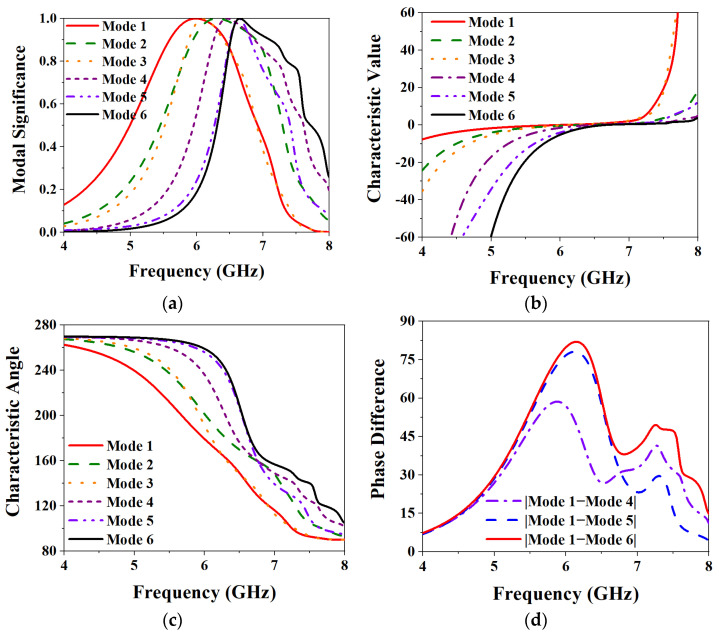
CMA results of metasurface layer: (**a**) MS; (**b**) CV; (**c**) CA; (**d**) Phase difference.

*CMA-based metasurface layer analysis.* Taking the first six main characteristic modes of the metasurface layer for analysis, Figure 4 shows the CMA results. It can be seen from Figure 4a that when the MS value of each mode is 1, all are around 5.9–6.6 GHz. In particular, the MS values of Mode 1 and Mode 6 are at 5.9 GHz and 6.6 GHz, respectively. Figure 4b shows the results of the CV analysis. The physical meaning of CV represents the power stored by the conductor when the radiated power of each characteristic current is a unit quantity. When the CV of a certain mode is smaller, it means that the radiation efficiency of the mode is higher. It can be seen from the figure that the CVs of each mode approach 0 at their MS value of 1, respectively, reaching the resonance state. Figure 4c,d show the analysis results of the characteristic angle (CA). The CA is used to characterize the phase angle between the characteristic current of a mode and its corresponding characteristic far-field from a physical point of view. The calculation formula is:

(2) $${\mathrm{CA}}_{n}{=180\text{°}\;-\;\tan}^{-1}\lambda_{n}$$

when the CA of a mode is equal to 180°, it means that the mode reaches a resonance state at that frequency. It can be seen from Figure 4c that the CA value of each characteristic mode at its resonance frequency point is close to 180°, reaching the resonance state. And Mode 2 and Mode 3 are relatively close to Mode 1, so the phase difference between them with Mode 1 is very small, and it is difficult to achieve the phase difference of 90° required by CP. So take Mode 4, Mode 5, and Mode 6 to make a phase difference with Mode 1, and observe the conditions for realizing CP as shown in Figure 4d. It can be seen that the phase difference between Mode 4 and Mode 5 with Mode 1 is 58.6° and 78.1° respectively, but the phase difference between Mode 6 and Mode 1 is 82°, which is closer to 90° required to achieve CP.

**Figure 5:**
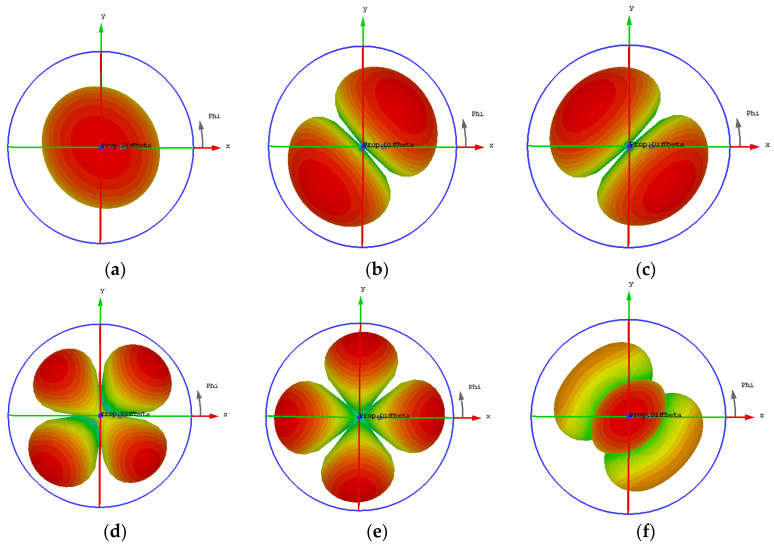
Far-field results for each characteristic mode at 6 GHz: (**a**) Mode 1; (**b**) Mode 2; (**c**) Mode 3; (**d**) Mode 4; (**e**) Mode 5; (**f**) Mode 6.

To study the radiation properties of the metasurface layer, the far-field results of each characteristic mode at 6 GHz are shown in Figure 5. It can be seen from the figure that Mode 1 and Mode 6, Mode 2, and Mode 3 are respectively orthogonal. The radiation directions of Mode 1 and Mode 6 are mainly along the z-axis. Although the radiation pattern of Mode 6 produces side lobes, the main radiation direction is still along the z-axis and has strong directivity, which proves that the current distribution is intensive in the middle of the metasurface layer. Although Mode 2 and Mode 3 are also orthogonal, their radiation direction is along two diagonal lines, and the maximum radiation intensity is mainly radiating at both sides. The radiation patterns of Mode 4 and Mode 5 are divided into four parts, respectively, without a uniform radiation direction, and the radiation intensity in the middle part is weak. If the slot-coupled feeding method is adopted, the slot part is placed in the middle part of the metasurface layer, and the orthogonal Mode 1 and Mode 6 will be excited to the greatest extent. Therefore, Mode 1 and Mode 6 are selected as the main radiation modes of the metasurface layer for further analysis.

**Figure 6:**
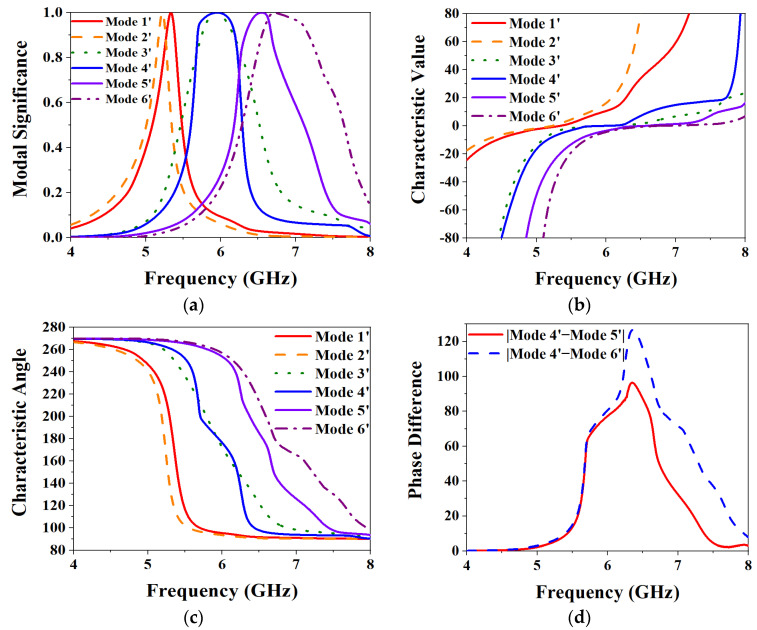
CMA results of the overall structure of the MIMO antenna element: (**a**) MS; (**b**) CV; (**c**) CA; (**d**) Phase Difference.

*CMA-based overall MIMO antenna element analysis.* Taking the first six most distinctive patterns of the overall structure for analysis, Figure 6 shows the CMA results of the overall MIMO element structure. From the MS in Figure 6a, it can be seen that the two slot modes, Mode 1′ and Mode 2′, generated by the slot layer, are located at 5.3 GHz and 5.2 GHz, respectively, and reach a maximum at their frequency. Although compared with the previous MS results for the slot layer, the resonance points of the two slot modes are shifted, but the shift rate is small. And after adding the metasurface layer, the potential bandwidth of the slot mode is broadened, and it still behaves as an LP mode. The remaining four characteristic modes are generated by the metasurface layer, among which Mode 4′ and Mode 5′ correspond to Mode 1 and Mode 6 of the metasurface layer, respectively. The frequencies at which the MSs of Mode 4′ and Mode 5′ reach their maximum values are 5.9 GHz and 6.5 GHz, respectively. According to the analysis of the metasurface layer alone above, only Mode 6′ is shifted by about 0.1 GHz. Figure 6b shows the CVs of each of the selected characteristic modes, all close to 0 at their resonant frequencies. Figure 6c is the CA curve of the six characteristic modes. It can be seen from the figure that the curves of Mode 4′ and Mode 3′ are very close, so if these two are selected as the characteristic modes for realizing CP, the result of the phase difference will be very small. So Figure 6d shows the results of the phase difference of Mode 5′ and Mode 6′ with respect to Mode 4′. As can be seen from the figure, the maximum value of the difference between Mode 4′ and Mode 5′ is 96.4°, but the maximum value of the phase difference between Mode 4′ and Mode 6′ reaches 126.7°, which is far from the condition of realization of CP.

The total characteristic current can be expressed as a linear superposition of a series of characteristic currents in a perfect electrical conductor (PEC); the formula is:

(3) $$J_{total}=\underset{n}{\sum }\alpha_{n}J_{n}$$

where $J_{n}$ represents the characteristic current and the $\alpha_{n}$ represents the weight coefficient of the *n*-th mode, also known as the modal weighting coefficient (MWC). When the value of the MWC of a certain mode is larger, it means that the mode can be excited to the greatest extent after adding the corresponding excitation structure in the appropriate position. From [21], MWC is also an important condition for realizing CP radiation, which must be satisfied:

(4a) $$∠\alpha_{1}-∠\alpha_{2}=\text{±}\pi /2$$

(4b) $$\left|\alpha_{1}\right|=|\alpha_{2}|.$$

**Figure 7:**
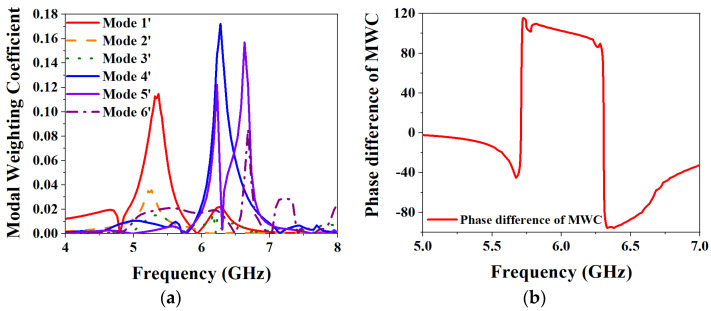
MWC results of the overall MIMO antenna element: (**a**) MWC; (**b**) Phase difference of MWC.

Figure 7 shows the results of the MWC analysis of the overall antenna element structure. It can be seen from the figure that the selected characteristic modes are excited to different degrees, but the selected Mode 1′, Mode 4′ and Mode 5′ play the main role of radiation, and the maximum frequency points are located at 5.4 GHz, 6.3 GHz, and 6.6 GHz. The other modes are excited to a small extent, but the influence on the overall radiation of the antenna element can be ignored. Figure 7b shows the results of the phase difference of the MWCs of Mode 4′ and Mode 5′, and it can be seen that the two selected metasurface modes have the conditions to realize CP. Therefore, Mode 1′ is finally selected as the slot mode for realizing LP, and Mode 4′ and Mode 5′ are selected as the metasurface modes for realizing CP.

### 2.2. Measurement Results and Discussion

**Figure 8:**
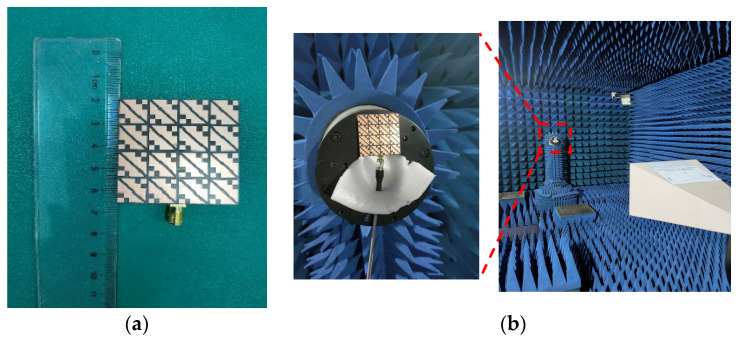
Fabricated MIMO antenna element and measurement environment: (**a**) Fabricated MIMO antenna element; (**b**) Anechoic chamber measurement environment.

**Figure 9:**
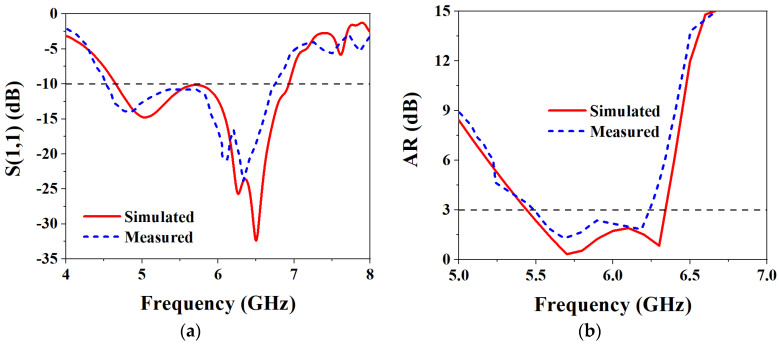
Simulated and measured S_11_ and ARBW results of the proposed MIMO antenna element: (**a**) S_11_; (**b**) ARBW.

**Figure 10:**
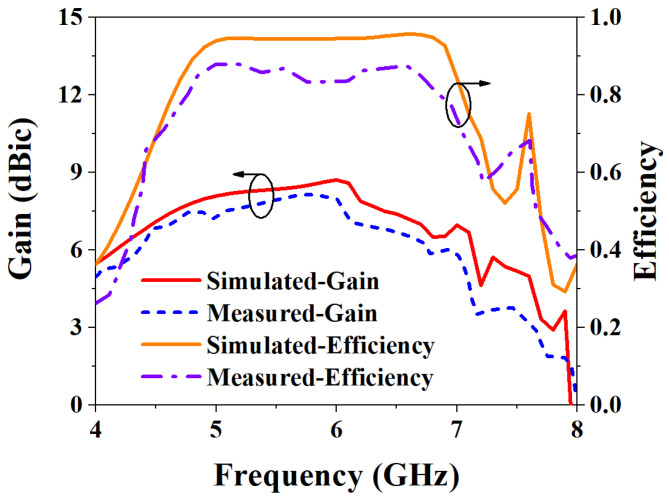
Simulated and measured gain and efficiency results of the proposed MIMO antenna element.

**Figure 11:**
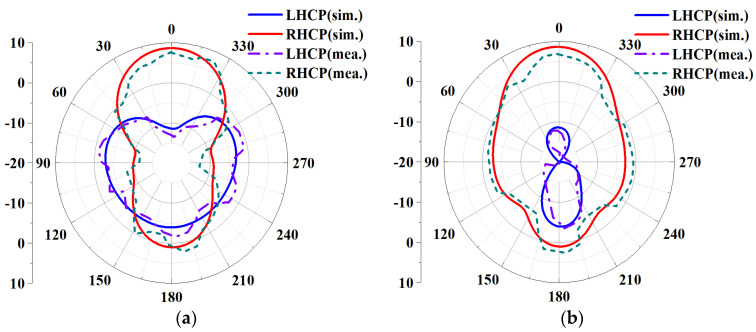
Comparing the simulated and measured radiation patterns of the proposed MIMO antenna element at 6 GHz: (**a**) Phi = 0°; (**b**) Phi = 90°.

Based on the above analysis of the designed MIMO antenna element, to verify the correctness of the analysis results, the designed MIMO antenna element is fabricated and measured. The fabricated object is shown in Figure 8a, and Figure 8b shows the measurement environment in the microwave anechoic chamber. By using the vector network analyzer of Rohde & Schwarz to measure and analyze the fabricated MIMO antenna element, the measured bandwidth results are shown in Figure 9. Figure 9a shows that the simulated −10 dB IMBW is 39.58% (4.62–6.9 GHz), the measured −10 dB IMBW is 38.93% (4.51–6.69 GHz), and Figure 9b shows the simulated 3 dB ARBW is 15.37% (5.4–6.31 GHz), and the measured 3 dB ARBW is 13.13% (5.48–6.25 GHz). Figure 10 shows the simulated and measured gain and efficiency, and the results show that the simulated and measured gain is 8.71 dBic and 8.25 dBic, respectively, and the simulated and measured efficiency are 95.6% and 88.1%, respectively. Figure 11 shows the simulated and measured radiation patterns when phi is 0° and 90°, respectively. The magnitudes of the results show that right-handed circular polarization (RHCP) is larger than left-handed circular polarization (LHCP) both when phi is 0° and 90°, indicating that the CP exhibited by the proposed MIMO antenna element is RHCP. Although there are some discrepancies between the measurement results and the simulation results, it is unavoidable because of errors caused by fabricating and some factors in the measurement environment in the anechoic chamber, but it does just have a little effect on the performance. Through the above processing and testing of the proposed MIMO antenna element, it is proved that the MIMO antenna element has the characteristics of wide bandwidth, CP, and high gain, so it has certain application prospects in biomedical.

## 3. Design and Analysis of MIMO Metasurface Antenna

MIMO antenna is the technology with the most development potential as the next-generation wireless communication technology. It is used to improve the capacity and reliability of the channel and can be regarded as a practical development direction to release the data rate limit of the single-input and single-output (SISO) system [7]. Based on the above in-depth analysis of the MIMO antenna element, it can be found that the proposed antenna element can work well in the 5 GHz band, achieving an excellent performance of wideband, CP, and high gain. Furthermore, to increase the data transmission rate and channel capacity, a two-port MIMO antenna is designed based on the proposed antenna element so that the applied wearable device is more suitable for future wireless communication of biomedical [22].

**Figure 12:**
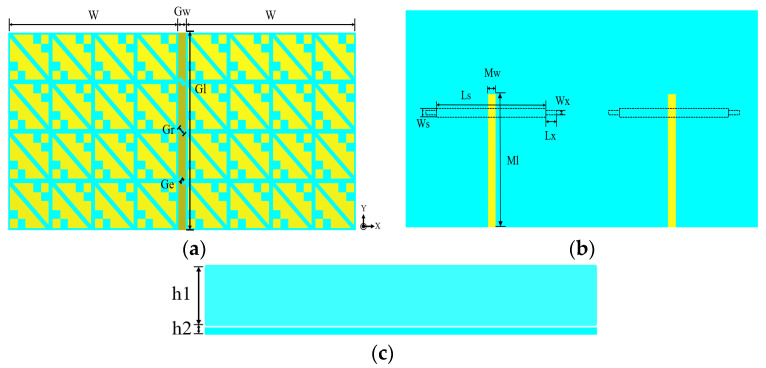
Structure of the two-port MIMO antenna: (**a**) Front view; (**b**) Back view; (**c**) Side view.

The structure of the proposed two-port wearable metasurface MIMO antenna is shown in Figure 12. The designed MIMO antenna is still a typical sandwich structure, with three layers of metal layers and two layers of the dielectric substrate, and the dielectric substrate still uses the PTFE ($\varepsilon_{r}$ = 2.65, tanδ = 0.0015). Under the condition that the size of the metasurface unit, the height of the dielectric substrate, the size of the slot layer, and the size of the microstrip line in the MIMO antenna element remain unchanged, a broadband wearable CP MIMO antenna with high isolation is designed. An isolation strip with a length of *Gl* = 44 mm and a width of *Gw* = 2 mm is added between the two antenna elements, and three small rectangles with a length of *Gr* = 2 mm and a width of *Ge* = 1 mm are etched in the isolation strip. In this way, the isolation between the two antennas is improved, thereby improving the overall performance of the MIMO antenna.

### 3.1. Isolation Analysis

**Figure 13:**
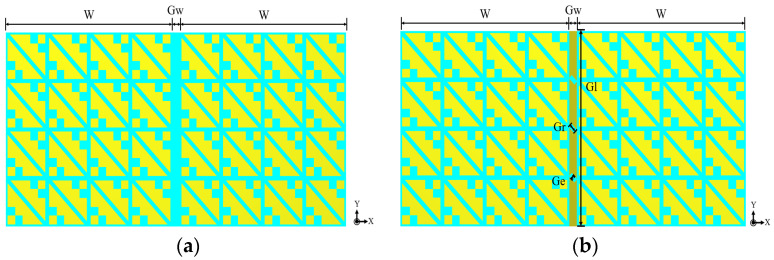
MIMO antenna without and with isolation strip: (**a**) Without isolation strip; (**b**) With isolation strip.

**Figure 14:**
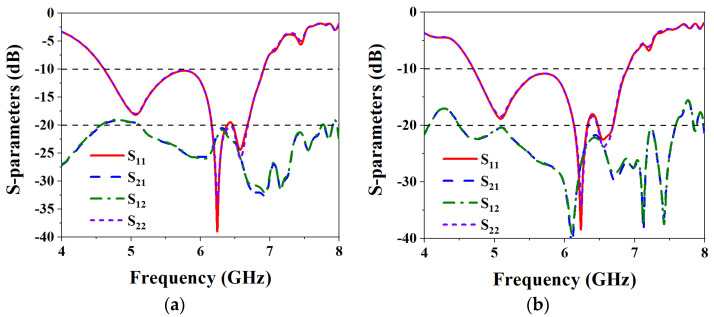
Simulated S-parameters of MIMO antenna without and with isolation strip: (**a**) Without isolation strip; (**b**) With isolation strip.

Figure 13 shows the structure diagram of the designed wearable MIMO antenna without adding an isolation strip and adding an isolation strip. The distance between the two antenna elements is *Gw*. Figure 14 shows the S-parameters curves without and with the addition of the isolation strip. It can be seen from Figure 14a that without adding an isolation strip, the isolation of the two elements is less than −19 dB in the working frequency band of the MIMO antenna. The results show that without adding isolation structures, even if the two antenna elements are not too far apart, they exhibit good isolation. This is because the designed metasurface units play a certain role in reducing isolation [23]. Although the isolation effect can also be achieved without adding an isolation strip, the isolation effect is still not so ideal, and it is difficult to obtain the excellent performance of the MIMO antenna. Figure 14b shows the S-parameters curves after adding an isolation strip to the metasurface layer of the MIMO antenna. It can be seen from the figure that the isolation of the two antenna elements in the working frequency band is less than −20.35 dB, which is 1.35 dB higher than that without the isolation strip. When the isolation strip is added, the MIMO antenna exhibits excellent isolation in the entire operating frequency band, demonstrating excellent MIMO antenna performance.

### 3.2. SAR Analysis

**Figure 15:**
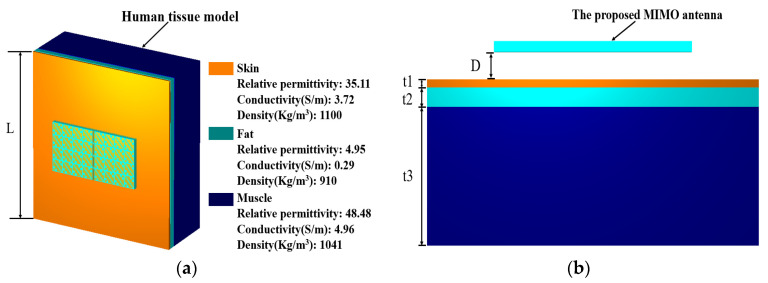
Human phantom model: (**a**) Perspective view; (**b**) Side view.

Figure 15 shows that the designed MIMO antenna is placed on the human tissue phantom model to observe the degree of harm to the human body when it is working. A standard three-layer human tissue phantom model is used, namely the skin layer, the fat layer, and the muscle layer, and the thicknesses of the three layers are *t*1 = 3 mm, *t*2 = 7 mm, and *t*3 = 50 mm, respectively [8]. The length of the model of human tissue is *L* = 150 mm, and considering the influence of the thickness of clothes in real life, the MIMO antenna is placed at a distance of *D* = 10 mm from the human body.

**Figure 16:**
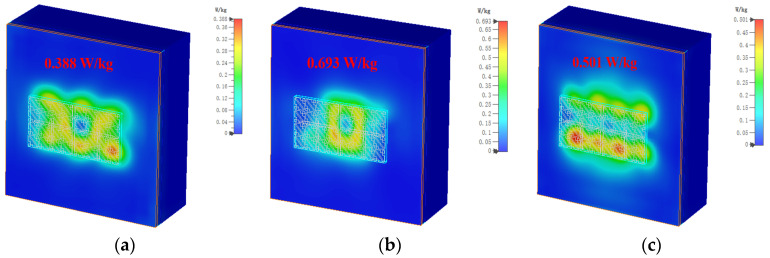
SAR results for simulated 1 g tissue at different frequencies: (**a**) 5 GHz; (**b**) 5.5 GHz; (**c**) 6 GHz.

**Figure 17:**
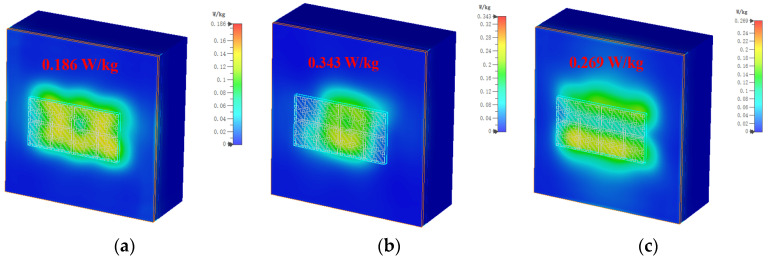
SAR results for simulated 10 g tissue at different frequencies: (**a**) 5 GHz; (**b**) 5.5 GHz; (**c**) 6 GHz.

In order to ensure that the designed antenna is safe for the human body when it is worn on the body, the specific absorption rate (SAR), an important parameter, is used to measure the level of electromagnetic waves emitted by the antenna being absorbed by the human body. The formula for calculating the specific absorption rate is:

(5) $$\mathrm{SAR}=\frac{\sigma {\left|E\right|}^{2}}{\rho }$$

where *σ* is the electrical conductivity of human tissue in S/m, *E* represents the effective electric field in V/m, and *ρ* denotes the mass density of human tissue in kg/m^3^. The US and EU have established standards for SAR, and the safe SAR values for 1 g and 10 g tissue are 1.6 W/kg and 2 W/kg, respectively [20]. To assess the SAR performance, the input power that the antenna will accept is set to 150 mW as a benchmark. Figure 16 shows the simulated 1 g tissue SAR values when the MIMO antenna is at different frequencies. It can be seen from the figure that the SAR values when the MIMO antenna is located at 5 GHz, 5.5 GHz, and 6 GHz are 0.388 W/kg, 0.693 W/kg, and 0.501 W/kg, respectively. Although the SAR maximum at 5.5 GHz reaches 0.693 W/kg, the SAR value for 1 g of human tissue is far below the US requirement of less than 1.6 W/kg. Figure 17 shows the SAR values at different frequencies when simulating a 10 g human tissue phantom model; the SAR values at 5 GHz, 5.5 GHz, and 6 GHz are 0.186 W/kg, 0.343 W/kg, and 0.269 W/kg, respectively. The SAR value under 10 g of human tissue has a very small degree of change, which is basically around 0.2 W/kg, and the maximum SAR value is 0.343 W/kg at 5.5 GHz. The simulated SAR values under 10 g of human tissue are all lower than the EU requirement of less than 2 W/kg. It is proved that the designed MIMO antenna exhibits low backward radiation and low SAR value when worn on the real human body, and the proposed antenna is harmless to the human body.

### 3.3. Measurement Results and Discussion

**Figure 18:**
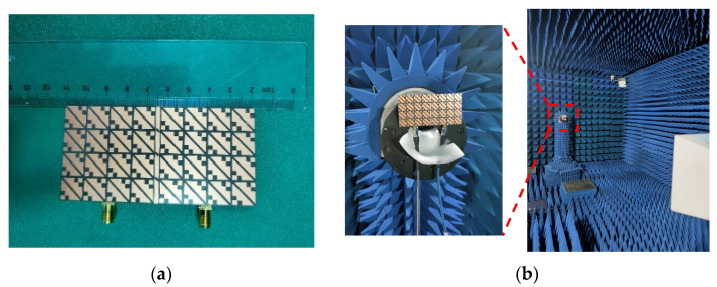
Antenna object and measurement environment: (**a**) Fabricated MIMO antenna; (**b**) Anechoic chamber measurement environment.

Based on the above simulation analysis of the performance of the MIMO antenna, then the MIMO antenna is fabricated, and its performance is analyzed. Figure 18a,b show the fabricated antenna and the measurement environment of the microwave anechoic chamber, respectively.

**Figure 19:**
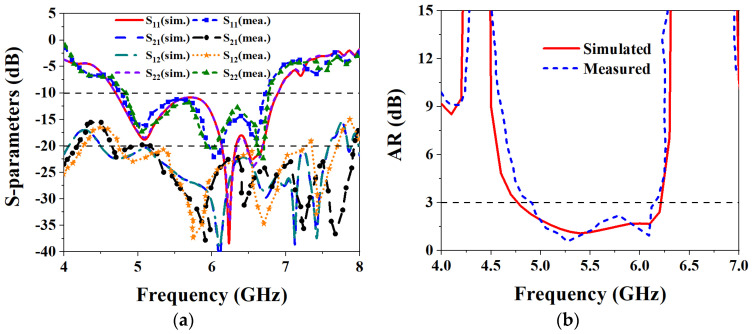
Comparison of simulated and measured results of S-parameters and ARBW: (**a**) S-parameters; (**b**) ARBW.

**Figure 20:**
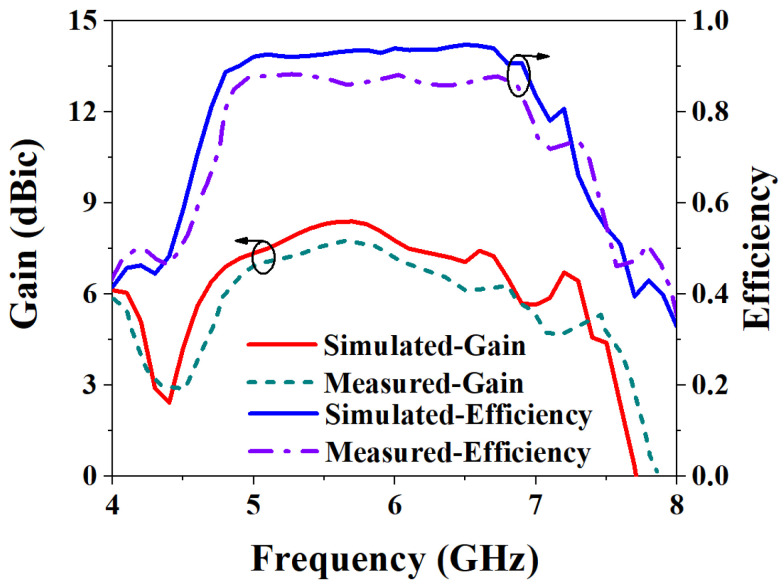
Comparison of simulated and measured results of the MIMO antenna gain and efficiency.

**Figure 21:**
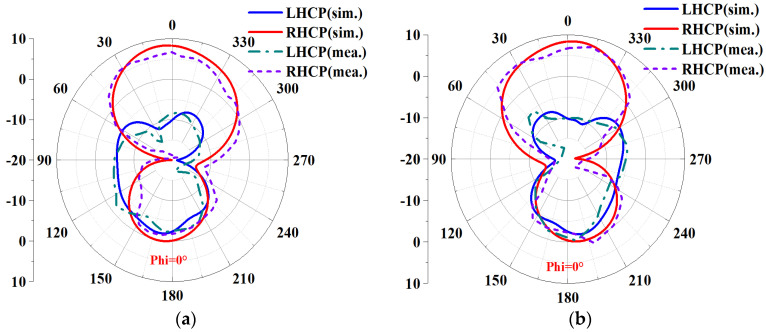

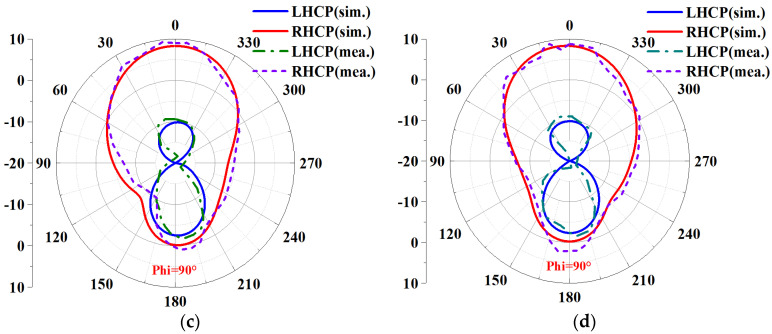
Simulated and measured radiation pattern of MIMO antenna at 5.6 GHz: (**a**) Port 1 at phi = 0°; (**b**) Port 2 at phi = 0°; (**c**) Port 1 at phi = 90°; (**d**) Port 2 at phi = 90°.

The S-parameters are measured using a Rohde & Schwarz vector network analyzer, and the results are shown in Figure 19a. It can be seen from the figure that the IMBW of −10 dB simulated by S_11_ and S_22_ of the MIMO antenna is 37.79% (4.7–6.89 GHz), and both S_21_ and S_12_ are less than −20.35 dB in the working frequency band, which satisfies the requirements of high isolation. The measured −10 dB IMBW for S_11_ and S_22_ is 34.87% (4.76–6.77 GHz). However, the measured S_21_ is less than −19.85 dB in the working frequency band; although it is about 0.15 dB different from the simulation result, overall, it still achieves high isolation performance. Figure 19b shows the simulated and measured ARBW, the simulated 3 dB ARBW is 26.59% (4.76–6.22 GHz), and the measured 22.94% (4.9–6.17 GHz), which is 3.65% narrower than the simulated ARBW result. Figure 20 shows a comparison of simulated and measured efficiency and gain. The simulated and measured maximum efficiency is 94.56% and 88.34%, respectively, and the maximum gain is 8.4 dBic and 7.95 dBic, respectively. Figure 21 shows the simulated and measured radiation patterns at 5.6 GHz when the two ports are separately excited. It can be seen from the figure that no matter whether phi is 0° or 90°, the pattern presented when one of the ports is excited alone is RHCP greater than LHCP. Although the measured pattern will deviate from the simulated results, it can be seen that the overall radiation of the antenna is very excellent.

#### 3.3.1. ECC and DG Characterization

For a comprehensive and in-depth understanding of the performance of the proposed MIMO antenna, there are different meaningful parameters to measure. In MIMO systems, the ECC is used to measure the correlation between different ports of the antenna. Use the following formula to evaluate the two-port correlation:

(6) $$\mathrm{ECC}=\frac{\left|S_{11}^{*}S_{12}{\;+\;S}_{21}^{*}S_{22}\right|}{(1\;-\;{\left|S_{11}\right|}^{2}\;-\;{\left|S_{21}\right|}^{2})(1\;-\;{\left|S_{22}\right|}^{2}\;-\;{\left|S_{12}\right|}^{2})}$$

where the S-parameters are the values of the different ports of the MIMO antenna. Usually, ECC is required to be less than 0.5. Another important parameter is diversity gain (DG), which is related to ECC and the calculation formula for DG is as follows:

(7) $$\mathrm{DG}=10\sqrt{{1\;-\;\mathrm{ECC}}^{2}}$$

**Figure 22:**
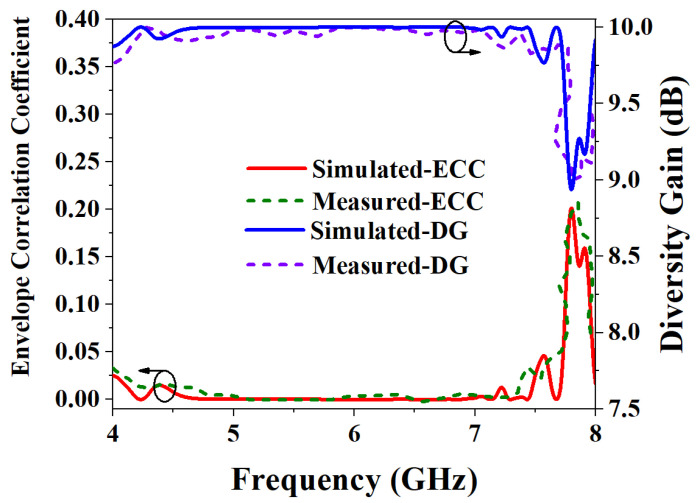
Simulated and measured results of ECC and DG.

Generally, DG is required to be greater than 9.95 dB. Figure 22 shows the simulation and measurement results of ECC and DG. It can be seen from the figure that ECC is less than 0.007 in the entire working frequency band, which meets the requirement that ECC is less than 0.5. At the same time, the ECC can also show that the isolation degree of the two ports of the designed MIMO antenna is very high. In addition, the maximum value of the simulated DG in the working frequency band of the antenna is 9.99 dB, and the measured DG is more than 9.98 dB, which meets the requirement of greater than 9.95 dB.

#### 3.3.2. Multiplexing Efficiency and Total Active Reflection Coefficient

Multiplexing efficiency (ME) is a method that provides a simple way to comprehensively describe the effect of antennas on the channel, including the efficiency, efficiency imbalance, and correlation between receive antennas. The formula for calculating the reuse efficiency is as follows:

(8) $$\eta_{\mathrm{Mux}}=\sqrt{{\left(1\;-\;|\rho \right|}^{2}{)\eta }_{1}\eta_{2}}$$

where $\eta_{\mathrm{Mux}}$ represents ME, and $\eta_{1}$ and $\eta_{2}$ represent the efficiencies of element-1 and element-2, respectively, and ρ represents ECC. The parameter total active reflection coefficient (TARC) takes this influence into account and is defined as the square root of the ratio of the total incident power to the reflected power in the entire MIMO system and the calculation formula is:

(9) $$\mathrm{TARC}=\frac{\sqrt{{{(S}_{11}{+S}_{22})}^{2}{+(S}_{21}{+S}_{12}{)}^{2}}}{\sqrt{2}}$$

**Figure 23:**
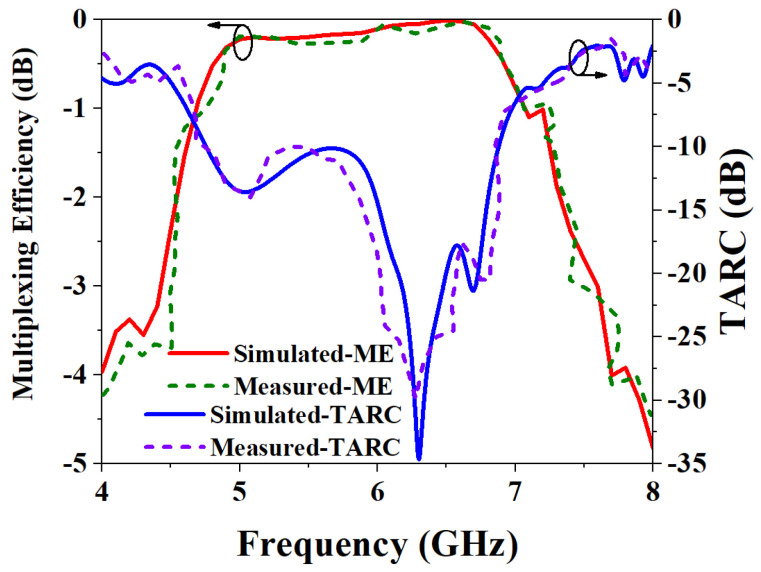
Simulated and measured results of ME and TARC.

Usually, TARC is required to be less than 0 dB. The simulated and measured results of ME and TARC are shown in Figure 23. The results of the simulation and measurement of ME are highly consistent, and both are less than 0 dB, which meets the requirements. It can be seen from the TARC results of the MIMO antenna that both the simulated and measured TARC of the antenna are less than 0 dB, which also shows that the two ports have very little influence on each other when they work simultaneously.

#### 3.3.3. Mean Effective Gain and Channel Capacity Loss

Analyzing the mean effective gain (MEG) of a MIMO antenna can help identify the fading environment of a MIMO antenna. In MIMO antennas, MEG can be defined as the ratio of the average power received from the antenna in some arbitrary direction to the average incident power to the antenna in the same direction. It shows that the influence of the wireless communication environment on diversity has been considered, and its calculation formula is below:

(10a) $${\mathrm{MEG}}_{1}=0.5_{\eta 1,\;\mathrm{rad}}=0.5[1-{\left|S_{11}\right|}^{2}-{\left|S_{12}\right|}^{2}]$$

(10b) $${\mathrm{MEG}}_{2}=0.5_{\eta 2,\;\mathrm{rad}}=0.5[1-{\left|S_{12}\right|}^{2}-{\left|S_{22}\right|}^{2}]$$

**Figure 24:**
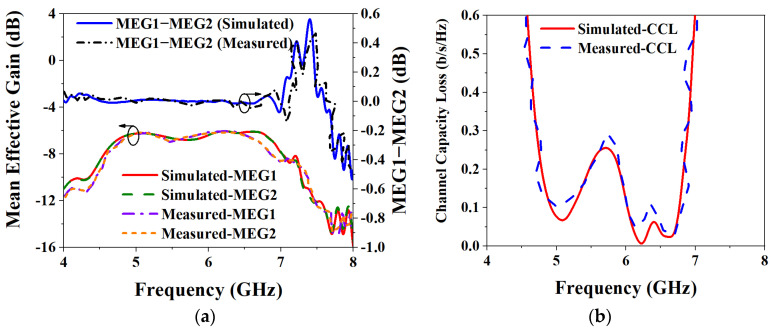
Simulated and measured results of MEG and CCL: (**a**) MEG; (**b**) CCL.

In the above equations, the S-parameters are the coefficients of each port, and the MEG ratio of the MIMO antenna is required to be within ±3 dB to provide good characteristics. When multiple ports in a MIMO antenna work at the same time, they will have a certain impact on each other’s performance. Figure 24a shows the results of the MEG analysis and both the simulated and measured MEGs of the two ports are less than −6 dB. In addition, the ratio of the two MEGs is within ±0.02 dB over the entire operating frequency band. The MEG results are shown to meet the specified specification at ±3 dB, which also shows that the wireless communication environment has little effect on the performance of the MIMO antenna when it is operating.

Channel capacity loss (CCL) is an important performance index in the MIMO antenna system, which is used to characterize the capability of the MIMO channel. The definition formula of CCL is:

(11a) $${\mathrm{CCL}=-\log}_{2}{\;\det(\Psi }_{\mathrm{ant}})$$

where $\Psi_{\mathrm{ant}}$ is a 2 × 2 correlation matrix, as shown in the following formula:

(11b) $$\Psi_{\mathrm{ant}}=\left[\begin{array}{cc} \rho_{11} & \rho_{12}\\ \rho_{21} & \rho_{22} \end{array}\right]$$

where the formula for calculating the elements in the matrix is:

(11c) $$\rho_{\mathrm{ii}}=1-({\left|S_{\mathrm{ii}}\right|}^{2}+{\left|S_{\mathrm{ij}}\right|}^{2})$$

(11d) $$\rho_{\mathrm{ij}}{=-(S}_{\mathrm{ii}}^{*}S_{\mathrm{ij}}{+S}_{\mathrm{ji}}^{*}S_{\mathrm{jj}})$$

where items *i* and *j* represent 1 or 2, respectively. Generally, CCL is required to be less than 0.4 b/s/Hz in its operating frequency band. The CCL simulation and measurement results of the MIMO antenna are shown in Figure 24b. From the results, the simulated CCL of the MIMO antenna in its operating frequency band is less than 0.25 b/s/Hz, and the measured CCL is less than 0.29 b/s/Hz; both simulation and measurement meet the requirements of less than the specified 0.4 b/s/Hz, which meets the requirements of practical applications.

#### 3.3.4. S-Parameters for Bodily Attachments and Performance Comparison

**Figure 25:**
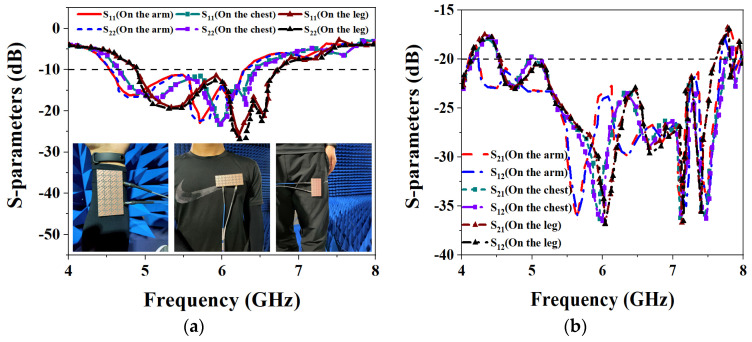
Measured S-parameters for placing the MIMO antenna at different parts of the human body: (**a**) S_11_ and S_22_; (**b**) S_21_ and S_12_.

**Figure 26:**
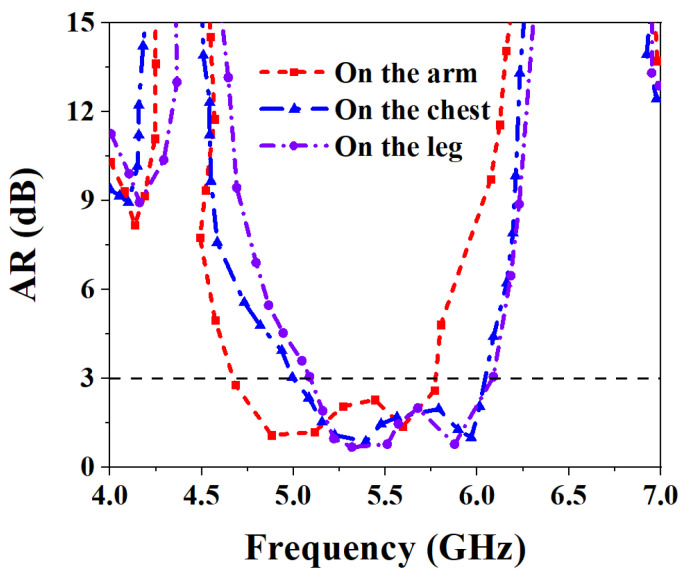
Measured ARBW of MIMO antenna placed at different parts of the human body.

For the diverse applications of the wearable antenna on the human body, the antenna was tested on the arm, chest, and leg. Figure 25 shows the measurement results of the S-parameters when the proposed MIMO antenna is worn on different parts of the human body. As can be seen from the figure, when worn on the arm, the measured IMBW of −10 dB for S_11_ and S_22_ is 32.47% (4.54–6.3 GHz), and both S_21_ and S_12_ are less than −20 dB in their operating frequency bands, meet the requirements of high isolation. When the proposed MIMO antenna is worn on the chest, the measured IMBW of −10 dB for S_11_ and S_22_ is 32.82% (4.61–6.42 GHz), but its S_21_ and S_12_ are less than −19.77 dB in its operating band, 0.23 dB away from the ideal isolation requirement. When the antenna is worn on the leg, the measured IMBW of −10 dB for S_11_ and S_22_ is 31.87% (4.88–6.73 GHz), and the S_21_ and S_12_ are both less than −20 dB in the operating frequency band, which is good isolation performance. Figure 26 shows the axial ratio results when worn on different body parts. It can be seen that the 3 dB ARBWs on the arm, chest and leg are 21.5% (4.65–5.77 GHz), 19.4% (4.98–6.05 GHz), and 17.43% (5.08–6.05 GHz), a wide ARBW is obtained and also achieve excellent CP performance.

Table 2 compares the performance of some wearable MIMO antennas published in recent years. It can be seen from the table that the ARBW of 3 dB in this paper is 5.25% smaller than that in [24], but the IMBW in this paper is better than it in terms of gain and isolation. Although the IMBW of −10 dB in [25] is the widest, this antenna only realizes the LP, and the gain and isolation are lower than those in this paper. The gain of [26] is the highest in the table, but its antenna size is large, only LP is achieved, and the IMBW is narrow. The measured isolation of the MIMO antenna in this paper is less than 19.85 dB in the working frequency band. Compared with other literature in the table, the isolation may not be particularly high, but other performance parameters are superior to other literature. Although the measurement results may be different due to fabricated errors and cable losses in the measurement environment, the impact on the overall performance of the antenna is very small. When the proposed wearable antenna is applied in reality, its performance may be affected by noise or interference, and the impact on antenna performance can be minimized by the circuitry and software in the communication system. Through the above measurement and analysis of the fabricated MIMO antenna, the new MIMO antenna proposed in this paper has the performance of CP, wide IMBW and ARBW, low ECC, and high gain, which is generally better than the antenna performance of related articles published in recent years, so the proposed MIMO antenna has a certain potential application in biomedical.

## 4. Conclusions

In this paper, a novel wearable metasurface MIMO antenna for 5 GHz WBAN is proposed. The principle of LP to CP conversion is explained by using CMA to analyze the different layers of the MIMO antenna element in detail. A new symmetric crown-shaped metasurface unit is proposed, and the polarization conversion is realized by using slot-coupled feeding. The dimensions of the proposed MIMO antenna element and the overall MIMO antenna are 0.81λ_0_ × 0.81λ_0_ × 0.07λ_0_ and 1.67λ_0_ × 0.81λ_0_ × 0.07λ_0_ at 5.6 GHz, respectively. The measurement and analysis confirm that the proposed MIMO antenna has a -10 dB IMBW of 34.87% (4.76–6.77 GHz) and a 3 dB ARBW of 22.94% (4.9–6.17 GHz). By adding an isolation strip, an isolation greater than 19.85dB is obtained, and a high gain of 7.95 dBic is achieved. The ECC is less than 0.007, with the maximum DG greater than 9.98 dB, and both ME and TARC are less than 0 dB. The MEG ratio is between ±0.02, and the CCL is less than 0.29 b/s/Hz. Comparing the SAR of the MIMO antenna in the human tissue phantom model of 1g and 10g at different frequencies, it is confirmed that the proposed antenna meets the requirements of international standards and is harmless to the human body. The proposed wearable metasurface MIMO antenna achieves wide IMBW, ARBW, high gain, high isolation, and excellent MIMO properties, and it is expected to find potential applications in various wearable biomedical devices.

## Figures and Tables

**Table 1:** Specific parameters of MIMO antenna element (in millimeters).

Parameter	Value (mm)	Parameter	Value (mm)
*W*	44	*S*	1
*h*_1_	3.5	*Ls*	30
*h*_2_	0.5	*Ws*	2
*W_p_*	10	*Lx*	3
*Wc*	2	*Wx*	1
*Lr*	14.4	*Ml*	27
*Wr*	1	*Mw*	2

**Table 2:** Comparison of wearable MIMO antenna performance in recent years.

References	*f*_0_ (GHz)	$\mathrm{Size}\;(\lambda_{0}^{3})$	Polarization (LP/CP)	−10 dB IMBW (%)	3 dB ARBW (%)	Gain (dBi)	Isolation (dB)	Elements
[14]	3.8	0.39 × 0.39 × 0.013	CP	26.32	37.84	3.45	24	2
[15]	5.2	0.64 × 0.24 × 0.26	CP	18.3	18.3	5.8	22	2
[22]	4.7	0.48 × 1.34 × 0.05	LP	20.5	-	7.83	25	3
[24]	3.75	0.46 × 0.38 × 0.01	CP	28.19	28.19	2.5	15	2
[25]	4.6	0.64 × 0.48 × 0.025	LP	58.56	-	3.5	17.5	4
[26]	5.6	2.53 × 1.0 × 0.05	LP	5.1	-	13.8	13.08	5
[27]	5.7	1.09 × 0.61 × 0.03	LP	3.6	-	6.43	25	2
[28]	5	3.33 × 1.16 × 0.026	CP	24.23	6.06	5.4	17	2
[29]	5	0.5 × 0.5 × 0.013	LP	28.8	-	4.8	15.4	4
[30]	5.8	0.53 × 1.87 × 0.03	CP	11.85	1.65	5.34	33	2
[31]	8.8	3.2 × 2.4 × 0.4	LP	14.5	-	7	32	4
[32]	4.9	0.88 × 0.44 × 0.02	LP	4.63	-	4.8	35	2
[33]	5.2	0.95 × 0.71 × 0.05	CP	8.3	8.3	6.2	26	2
[34]	5.6	0.43 × 0.93 × 0.03	CP	20.69	6.31	6	37	2
[35]	3.7	1.85 × 0.93 × 0.04	CP	10.9	4.12	3.2	20	4
Proposed	5.6	1.67 × 0.81 × 0.07	CP	34.87	22.94	7.95	19.85	2

## Data Availability

Not applicable.
